# The tyrosine phosphatase PTPRO sensitizes colon cancer cells to anti-EGFR therapy through activation of SRC-mediated EGFR signaling

**DOI:** 10.18632/oncotarget.2458

**Published:** 2014-10-11

**Authors:** Layka Abbasi Asbagh, Iria Vazquez, Loredana Vecchione, Eva Budinska, Veerle De Vriendt, Maria Francesca Baietti, Mikhail Steklov, Bart Jacobs, Nicholas Hoe, Sharat Singh, Naga-Sailaja Imjeti, Pascale Zimmermann, Anna Sablina, Sabine Tejpar

**Affiliations:** ^1^ Laboratory of Molecular Digestive Oncology, Department of Oncology, KU Leuven, Leuven, Belgium; ^2^ Laboratory for Mechanisms of Cell Transformation, VIB Center for the Biology of Disease, VIB, Belgium; ^3^ Center for Human Genetics, KU Leuven, Leuven, Belgium; ^4^ Institute of Biostatistics and Analyses, Faculty of Medicine, Masaryk University, Brno, Czech Republic; ^5^ Prometheus Laboratories, San Diego, CA, USA; ^6^ Centre de Recherche en Cancérologie de Marseille (CRCM), Inserm, U1068-CNRS UMR7258, Aix-Marseille Université, Institut Paoli-Calmettes, Marseille, France

**Keywords:** EGFR, PTPRO, phosphatase, SRC kinase, EGFR inhibitor, colon cancer

## Abstract

Inappropriate activation of epidermal growth factor receptor (EGFR) plays a causal role in many cancers including colon cancer. The activation of EGFR by phosphorylation is balanced by receptor kinase and protein tyrosine phosphatase activities. However, the mechanisms of negative EGFR regulation by tyrosine phosphatases remain largely unexplored. Our previous results indicate that protein tyrosine phosphatase receptor type O (PTPRO) is down-regulated in a subset of colorectal cancer (CRC) patients with a poor prognosis. Here we identified PTPRO as a phosphatase that negatively regulates SRC by directly dephosphorylating Y416 phosphorylation site. SRC activation triggered by PTPRO down-regulation induces phosphorylation of both EGFR at Y845 and the c-CBL ubiquitin ligase at Y731. Increased EGFR phosphorylation at Y845 promotes its receptor activity, whereas enhanced phosphorylation of c-CBL triggers its degradation promoting EGFR stability. Importantly, hyperactivation of SRC/EGFR signaling triggered by loss of PTPRO leads to high resistance of colon cancer to EGFR inhibitors. Our results not only highlight the PTPRO contribution in negative regulation of SRC/EGFR signaling but also suggest that tumors with low PTPRO expression may be therapeutically targetable by anti-SRC therapies.

## INTRODUCTION

Epidermal growth factor receptor (EGFR) plays a crucial role in the regulation of cellular homeostasis in both vertebrates and invertebrates by coordinating cell growth and proliferation. In *C. elegans* and *Drosophila*, the EGFR pathway controls the development of vulva and several other organs at different stages of embryogenesis, whereas in vertebrates EGFR signaling controls organogenesis of multiple epithelial tissues [1, 2]. EGFR hyperactivation is commonly observed in multiple types of epithelial cancers including colon cancer [3]. Multiple mechanisms of EGFR up-regulation, including amplification and activating mutations of the *EGFR* gene as well as overexpression of EGFR and the receptor ligands, are well-characterized. More recent studies also highlight the importance of negative regulation in control of EGFR signaling [4]. Nonetheless, the contributions of negative EGFR regulators are still underestimated, although understanding of their activities might form the foundation for a more effective anti-cancer approach.

Genetic screens in *C. elegans* have identified several negative regulators of EGFR including the E3 ubiquitin ligase SLI-1 (c-CBL) and the tyrosine phosphatase SCC-1, a R3 subtype of receptor-type protein tyrosine phosphatases (RPTPs) [5]. The *Drosophila* orthologs of R3 family members, Ptp4E and Ptp10D, have also been shown to negatively regulate EGFR signaling [6, 7]. Loss of both Ptp4E and Ptp10D results in large bubble-like cysts in tracheal branches, a phenotype commonly observed due to EGFR hyperactivation [7].

In vertebrates RPTPs of the R3 subtype include vascular endothelial–protein tyrosine phosphatase (VE-PTP), density-enriched PTP–1 (DEP-1), PTPRO (GLEPP1), and stomach cancer–associated protein tyrosine phosphatase–1 (SAP-1). All of these enzymes share a similar structure with a single catalytic domain in the cytoplasmic region and fibronectin type III–like domains in the extracellular region [8]. Recent studies have revealed additional common features of these R3-subtype RPTPs. For instance, all members of the R3 family undergo tyrosine phosphorylation in their COOH-terminal region, and such phosphorylation promotes the binding of SRC family kinases (SFKs) [9]. Their striking structural and sequence similarity suggests that they might function through a common mechanism [10]. In fact, recent unbiased siRNA screen targeting each of known tyrosine phosphatases identified two R3 family members, DEP-1 and PTPRO, as negative EGFR regulators in human cells [11]. DEP-1 has been shown to directly dephosphorylate and thereby stabilize EGFR by hampering its ability to associate with the c-CBL ubiquitin ligase. PTPRO has also been identified among the top hits and proposed to contribute to regulation of EGFR signaling. However, no further functional validations have been performed in this study [11].

Anti-EGFR monoclonal antibodies (cetuximab and panitumumab) and small-molecule tyrosine kinase inhibitors (gefitinib and erlotinib) have been recently approved by the Food and Drug Administration (FDA) for the treatment of metastatic colorectal cancer and non-small-cell lung cancer (NSCLC), squamous-cell carcinoma of the head and neck, and pancreatic cancer [12, 13]. Despite their highly promising activity of EGFR inhibitors for cancer treatment, there is a large group of *CRC* patients that do not respond to anti-EGFR therapy. The most well-established mechanism of cetuximab resistance in CRC patients is oncogenic *KRAS* mutations. However, not all patients harboring *WT-KRAS* benefit from cetuximab treatment. There is accumulating evidence that resistance to anti-EGFR therapy develops due to the loss of negative regulators of EGFR signaling [4, 13].

To date, only few data have been published about the contribution of PTPRO in colon cancer. Recent gene expression analysis of 688 primary colon tumors revealed that *PTPRO* mRNA expression is strongly down-regulated in colon cancer patients with a poor prognosis [14]. In the present study, we found that loss of PTPRO expression is associated with increased resistance to EGFR inhibition and identified PTPRO as a novel negative regulator of EGFR signaling that functions through direct dephosphorylation of the SRC kinase.

## RESULTS

### PTPRO controls EGFR stability and phosphorylation at Y845

**Figure 1:**
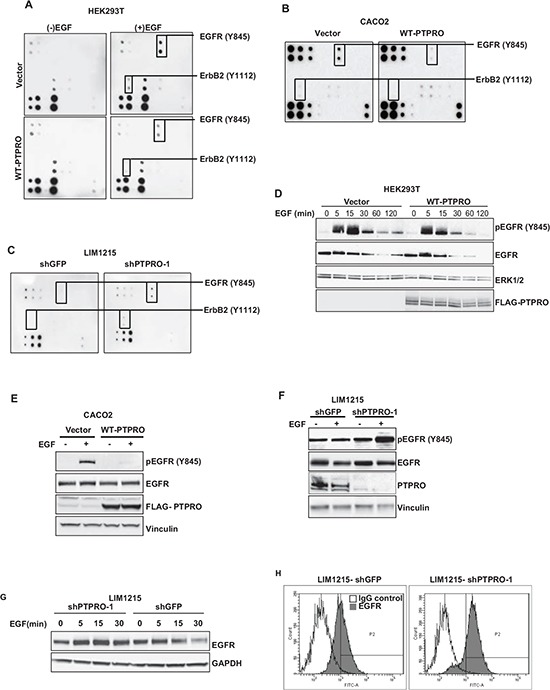
PTPRO contributes to EGFR regulation **A, B** The effect of PTPRO overexpression on EGFR phosphorylation. **(A)** Serum-starved (−EGF) HEK293T cells expressing either an empty vector, or WT-PTPRO were stimulated with EGF (100 ng/ml) for 15 minutes. Each dot represents specific tyrosine phosphorylation of EGFR family members at a specific site. **(B)** CACO2 cells expressing either an empty vector, or WT-PTPRO were serum-starved and then stimulated with EGF (20 ng/ml) for 15 minutes. **(C)** The effect of PTPRO suppression on EGFR phosphorylation. LIM1215 cells expressing the indicated construct were serum-starved overnight and stimulated with EGF (20 ng/ml) for 15 minutes. Each dot represents specific tyrosine phosphorylation of EGFR family members at a specific site. **D, E, F** Immunoblot analysis of total EGFR or EGFR phosphorylated at Y845. **(D)** Serum-starved HEK293T cells expressing the indicated constructs were stimulated with EGF (100 ng/ml) for different time points. **(E)** Serum-starved CACO2 cells expressing the indicated constructs were stimulated with EGF (20 ng/ml) for 15 min. **(F)** Serum-starved LIM1215 cells expressing either shPTPRO or shGFP were stimulated with EGF (20 ng/ml) for 15 min. **(G)** Immunoblot analysis of EGFR in LIM1215 cells expressing either shPTPRO, or shGFP. Cells were serum-starved overnight and stimulated with EGF (20 ng/ml) for the indicated time periods. **(H)** Flow cytometry analysis of cell-surface EGFR in LIM1215 cells expressing shRNAs against PTPRO or GFP. Cells were stained with FITC-conjugated anti-EGFR antibody or normal mouse IgG2 as a control.

A recent high-throughput siRNA screen suggested that PTPRO may be implicated in the regulation of EGFR signaling [11]. To elucidate the role of PTPRO in modulation of EGFR signaling, we assessed how PTPRO overexpression affects EGF-induced phosphorylation of several EGFR family members using The RayBio® EGFR Phosphorylation Antibody Array. In accordance to a recent report showing that ErbB2 is a direct substrate of PTPRO [15], we found that PTPRO overexpression in HEK293T cells diminished phosphorylation of ErbB2 at Y1112 upon EGF stimulation (Figure 1A). In addition to decreased phosphorylation of ErbB2 at Y1112, PTPRO overexpression also led to decreased EGFR phosphorylation at Y845 (Figure 1A). We observed similar results when we overexpressed WT-PTPRO in CACO2 colon cancer cell line, which does not express PTPRO (Figure 1B). In contrast, suppression of PTPRO in LIM1215 cells, which have high levels of PTPRO expression, resulted in increased EGFR phosphorylation at Y845 (Figure 1C). We were not able to detect EGF-mediated phosphorylation of other EGFR sites (Y992, Y1045, Y1068, Y1148, and Y1173) that were present on The RayBio® EGFR Phosphorylation Antibody Array (Figure 1A, B, and C).

To confirm the Antibody Array analysis, we assessed how PTPRO affects the kinetics of EGFR phosphorylation at Y845 upon EGF stimulation. We found that EGFR phosphorylation at Y845 was up-regulated in EGF-dependent manner in HEK293T cells expressing either an empty vector or WT-PTPRO. However, PTPRO overexpression led to more transient and decreased Y845-phosphorylation of EGFR (Figure 1D). We also observed a dramatic decrease of EGFR phosphorylation at Y845 upon EGF stimulation after WT-PTPRO overexpression in CACO2 cells (Figure 1E). In contrast, suppression of PTPRO expression in LIM1215 cells resulted in increased levels of EGFR phosphorylation at Y845 in response to EGF stimulation (Figure 1F). These results strongly indicate that PTPRO regulates EGFR phosphorylation at Y845.

We also found that PTPRO suppression inhibits down-regulation of EGFR expression triggered by EGF stimulation (Figure 1G). In addition, flow cytometry analysis revealed that PTPRO knockdown led to the increased EGFR levels on the plasma membrane (Figure 1H). Taken together, our data strongly indicate a crucial role of PTPRO in negative regulation of EGFR phosphorylation at Y845 as well as EGFR recycling and stability.

### PTPRO regulates EGFR phosphorylation at Y845 through direct activation of the SRC kinase

**Figure 2:**
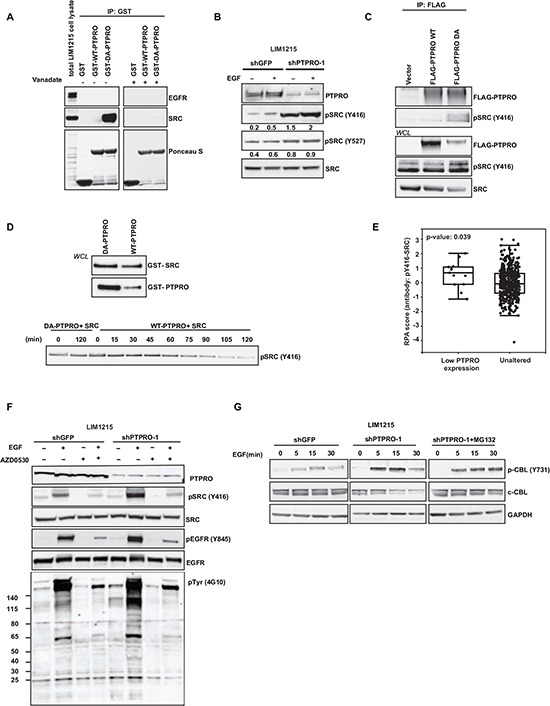
PTPRO controls EGFR by directly dephosphorylating the SRC kinase **(A)** PTPRO substrate-trapping assay. Cell lysates from pervanadate-treated LIM1215 cells were incubated with GSH-sepharose beads conjugated to GST-tagged catalytic domains of PTPRO (WT and DA) in the absence or presence of vanadate (1 mM). The pulled-down proteins were detected by immunoblotting with the indicated antibodies. The protein input was controlled with Ponceau S staining. **(B)** Immunoblot analysis of phospho-SRC and total SRC in serum-starved LIM1215 cells expressing shPTPRO or shGFP after 15 min of EGF stimulation (20 ng/ml). Levels of phosphorylated SRC normalized by total SRC expression were assessed by densitometry analysis using AIDA software. **(C)** Co-immunoprecipitation of Flag-tagged PTPRO (WT and DA) with SRC phosphorylated at Y416. 48 hours after overexpression with Flag-tagged forms of PTPRO (WT and DA), the indicated PTPRO constructs were pulled-down using anti-Flag agarose. Phospho-SRC (Y416) was detected by immunoblotting. **(D)** Purified GST-tagged catalytic domains of PTPRO (WT and DA) were incubated with GST-tagged recombinant active SRC kinase for different time points. Levels of SRC phosphorylated at Y416 were detected by immunoblotting. Equal loading of proteins was controlled by immunoblotting using GST specific antibody. **(E)** Boxplots of reverse phase protein array (RPPA) data showing phosphorylation status of SRC (Y416) in PTPRO down-regulated colorectal tumors. **(F)** Immunoblot analysis of the indicated proteins in LIM1215 cells expressing either shGFP or shPTPRO after treatment with AZD0530 (2μM) for 90 min in the presence or absence of EGF (20 ng/ml). **(G)** Immunoblot analysis of phospho-c-CBL (Y731) and c-CBL in LIM1215 cells expressing either shPTPRO or shGFP. Cells were serum-starved and stimulated with 20 ng/ml of EGF for the indicated time periods. MG-132 (10μM) was added to LIM1215-shPTPRO cells for 3 hours prior to EGF stimulation.

To test whether EGFR could be a direct substrate of PTPRO, we performed *in vitro* substrate trapping assay described in detail in [16, 17]. We incubated the intracellular domain of WT-PTPRO or a substrate-trapping PTPRO-DA mutant with LIM1215 cell lysates (Figure 2A). However, we did not detect any interaction with either WT-PTPRO, or PTPRO-DA mutant. Because a recent study demonstrated that EGFR and wild-type *Drosophila* ortholog of PTPRO (Ptp10D) form a complex through their extracellular domains [7], we suggested that extracellular domain of PTPRO could be crucial for the interaction with EGFR. Therefore, we assessed the interaction between a full-length WT-PTPRO or PTPRO-DA mutant and EGFR. In concordance with [7], we found that EGF stimulation triggered PTPRO binding to EGFR. However, we did not observe any stabilization of the interaction when we overexpressed substrate-trapping PTPRO-DA compared to WT-PTPRO (Supp. Figure 1). Together, these results suggest that EGFR is not a direct substrate of PTPRO.

On the other hand, several reports demonstrated that Y845 is not EGFR autophosphorylation site, instead EGFR phosphorylation at Y845 is regulated by the SRC kinase [18–20]. This suggests that PTPRO could affect EGFR phosphorylation by negatively modulating SRC activity. In fact, the *in vitro* substrate trapping assay revealed a strong interaction between the substrate-trapping PTPRO-DA mutant and the SRC kinase. To confirm these data, we used vanadate competition approach [16, 17]. Vanadate is a small phosphotyrosine mimetic molecule that competes with a phosphatase substrate for binding to the phosphatase catalytic site. We pre-treated recombinant WT-PTPRO or PTPRO-DA proteins with 1 mM vanadate before incubation with LIM1215 cell lysates. We found that pre-incubation with vanadate completely abolished the binding of PTPRO-DA mutant to SRC, strongly indicating that SRC is a direct substrate of PTPRO (Figure 2A).

A recent report showed that a truncated form of PTPRO, PTPROt, affects SRC phosphorylation at Y416 in B-cells [21]. Consistently with this study, we found that depletion of PTPRO in LIM1215 cells resulted in about 4-fold increase of SRC phosphorylated at Y416, whereas SRC phosphorylation at Y527 was only slightly increased upon PTPRO suppression (Figure 2B), suggesting Y416 site is the major site for PTPRO-dependent dephosphorylation. We also observed accumulation of SRC phosphorylated at Y416 when we overexpressed the substrate-trapping PTPRO-DA mutant (Figure 2C). Moreover, we found that PTPRO-DA trapping mutant formed a complex with SRC phosphorylated at Y416 (Figure 2C).

To confirm whether PTPRO directly dephosphorylates SRC at Y416, we performed *in vitro* dephosphorylation assay. Purified WT-PTPRO or catalytically inactive PTPRO mutant (PTPRO-DA) were incubated with recombinant active SRC protein for different periods of time. Immunoblotting analysis of Y416-phosphorylated SRC revealed that incubation with WT-PTPRO but not with PTPRO-DA led to a significant decrease of Y416-phosphorylated SRC (Figure 2D), further confirming that PTPRO directly dephosphorylates SRC at Y416.

Furthermore, data-mining of proteomics data from 461 primary colorectal tumor revealed that increased phosphorylation of SRC at Y416 (p=0.039) is observed in tumors with low *PTPRO* expression (cBioPortal, Cerami *et al*., 2012, http://cbioportal.org/public-portal/) (Figure 2E). These data corroborate that PTPRO directly dephosphorylates SRC at Y416.

SRC phosphorylation at Y416 plays a crucial role in the upregulation of its enzymatic activity [22–24], suggesting that PTPRO affects EGFR phosphorylation at Y845 by inhibiting SRC activity. To confirm that PTPRO affects EGFR phosphorylation at Y845 in a SRC-dependent manner, we assessed how inhibition of SRC activity would affect EGFR phosphorylation at Y845 in LIM1215 cells expressing either shGFP or shPTPRO. We found that treatment with AZD0530, a potent SRC inhibitor, completely blocked EGF-induced SRC activation in both cell lines. Importantly, AZD0530 abolished PTPRO-mediated difference in levels of EGFR phosphorylation at Y845 (Figure 2F). Taken together, these data indicate that PTPRO affects EGFR phosphorylation at Y845 by inhibiting SRC activity.

### SRC activation triggered by loss of PTPRO leads to c-CBL degradation

In addition to alterations in SRC and EGFR phosphorylation, PTPRO knockdown also affected tyrosine phosphorylation of multiple proteins in a SRC-dependent manner (Figure 2F), suggesting that several SRC substrates may contribute to PTPRO-dependent regulation of EGFR stability and trafficking (Figure 1 G, H). Given that SRC-mediated inhibition of c-CBL, the major ubiquitin ligase responsible for EGFR degradation, is one of the most well-established mechanisms by which SRC regulates EGFR stability [25], we examined whether PTPRO expression affects c-CBL phosphorylation and stability.

In line with the observation that PTPRO suppression led to SRC up-regulation (Figure 2B), we found that PTPRO attenuation resulted in increased c-CBL phosphorylation at Y731 (Figure 2G). Previous reports also demonstrated that SRC-mediated phosphorylation of c-CBL at Y731 promotes its auto-ubiquitination and proteasomal degradation [25, 26]. In fact, we observed down-regulation of c-CBL protein expression in PTPRO-depleted cells. On the other hand, treatment with the proteasomal inhibitor MG132 restored the c-CBL protein levels in PTPRO-knockdown cells (Figure 2G), suggesting that loss of PTPRO triggers proteasomal degradation of c-CBL by activating SRC activity. Given that c-CBL down-regulation suppresses EGF-mediated EGFR degradation, but facilitates its recycling [25], this indicates that PTPRO may regulate both EGFR stability and recycling by affecting SRC-mediated c-CBL phosphorylation at Y731.

In addition, SRC has been implicated in phosphorylation of a number of vesicular trafficking proteins such as clathrin, dynamin, caveolin that could also be in involved in modulation of EGFR trafficking [27–29]. Therefore, additional SRC substrates could contribute to the regulation of EGFR internalization and degradation.

### PTPRO inhibits EGF-dependent MAPK pathway activation

**Figure 3:**
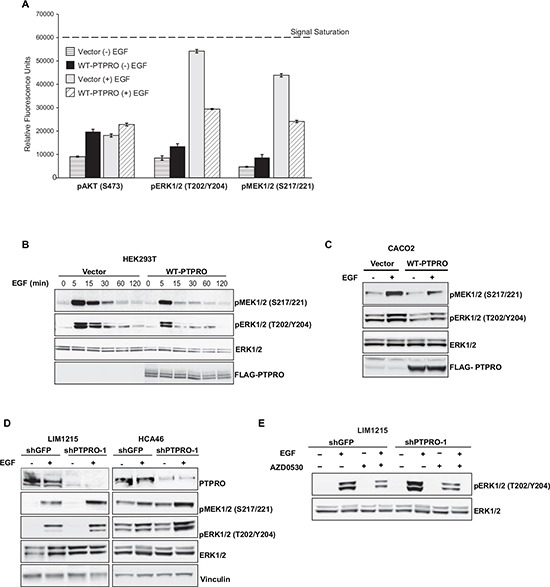
PTPRO inhibits EGF-dependent MAPK pathway activation **(A)** Phosphorylation status of the indicated kinases measured by *Collaborative Enzyme Enhance Reactive (CEER)* immunoassay after overexpression of WT-PTPRO. Serum starved cells expressing PTPRO or an empty vector were analyzed after EGF stimulation (100 ng/ml) for 15 min. The results are expressed as mean ± s.e.m. for two independent experiments. **(B)** Immunoblot analysis of phospho-ERK1/2 and MEK1/2 in serum-starved HEK293T cells expressing an empty vector or WT-PTPRO at different time points after EGF stimulation (100 ng/ml). **(C)** Immunoblot analysis of phospho-ERK1/2 and MEK1/2 in serum-starved CACO2 cells expressing an empty vector or WT-PTPRO after EGF stimulation (20 ng/ml) for 15 min. **(D)** Immunoblot analysis of phospho-ERK1/2 and MEK1/2 in LIM1215 and HCA46 cells expressing either shPTPRO or shGFP. Cells were serum-starved and stimulated with EGF (20ng/ml) for 15 min. **(E)** Immunoblot analysis of phospo-ERK1/2 and total ERK1/2 in LIM1215 cells expressing the indicated vectors after treatment with AZD0530 (2μM) for 90 min in the presence or absence of EGF (20 ng/ml).

Suppression of the SRC/EGFR pathway by PTPRO suggests its role in negative regulation of MAPK and PI3K/Akt signaling cascades that are two major pathways downstream of EGFR. We assessed the impact of PTPRO overexpression on phosphorylation levels of MAPK and Akt kinases, using the CEER immunoassay platform, a highly sensitive antibody-capture proximity-based immune-microarray [30]. We found that PTPRO overexpression significantly decreased EGF-induced phosphorylation of both ERK1/2 and MEK1/2 (Figure 3A). On the other hand, we did not observe any difference in Akt phosphorylation in EGF-stimulated cells expressing either an empty vector or Flag-tagged PTPRO. These results corroborate prior observations that EGFR engages multiple downstream pathways in ways that are context dependent (reviewed in [31]).

We confirmed the results of the CEER immunoassay by immunoblotting. Analysis of MEK1/2 and ERK1/2 phosphorylation kinetics upon EGF stimulation revealed that PTPRO overexpression dramatically reduced EGF-induced phosphorylation of both ERK1/2 and MEK1/2 (Figure 3B). We also observed decreased ERK1/2 and MEK1/2 phosphorylation after PTPRO overexpression in CACO2 cell line (Figure 3C). In contrast, suppression of PTPRO in LIM1215 and HCA46 colon cancer cells led to enhanced phosphorylation of the MAPK kinases (Figure 3D).

We next confirmed that PTPRO affects the MAPK signaling by modulating SRC activity. Indeed, we found that SRC inhibition by AZD0530 treatment completely abolished PTPRO-mediated difference in ERK1/2 phosphorylation (Figure 3E). Taken together, these results indicate that PTPRO controls of the MAPK signaling pathway in colorectal cancer cells by regulating SRC activity.

### Low PTPRO expression leads to increased resistance of colon cancer cells to EGFR inhibitors

**Figure 4:**
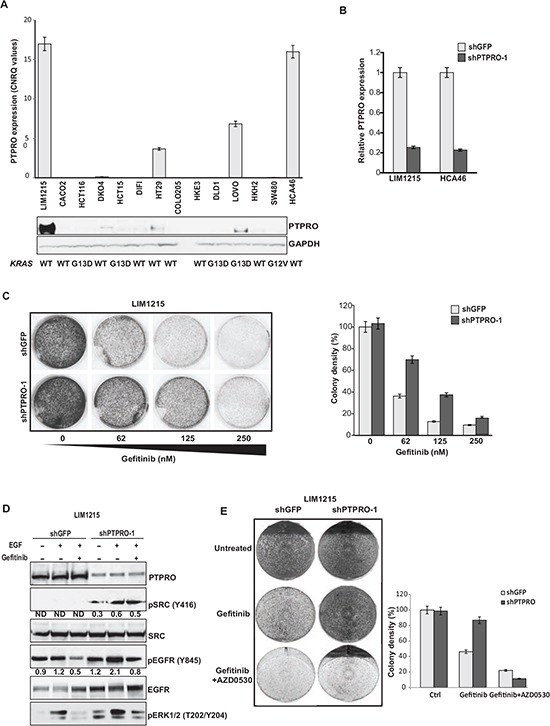
Resistance to gefitinib is associated with increased activation of EGFR and SRC in cells with low PTPRO expression **(A)** qRT-PCR and immunoblot analyses of PTPRO expression in a panel of human CRC cell lines. **(B)** qRT-PCR analysis of *PTPRO* mRNA expression in LIM1215 and HCA-46 cells expressing shRNAs against GFP or PTPRO. **(C)** Colony formation assay of LIM1215 cells expressing shRNAs targeting PTPRO or GFP treated with increasing concentrations of gefitinib. **(D)** Immunoblot analysis of the indicated proteins in LIM1215 cells expressing either shGFP or shPTPRO after treatment with gefitinib (1μM) for 1hr in the presence or absence of EGF (20 ng/ml). Levels of phosphorylated SRC and EGFR normalized by total SRC or EGFR expression, respectively, were assessed by densitometry analysis using AIDA software. **(E)** Colony formation assay of LIM1215 cells expressing shGFP or shPTPRO treated with gefitinib (62nM) or a combination of gefitinib (62nM) and AZD0530 (1 μM). **A, B, C** The results are expressed as mean ± s.e.m. for three independent experiments. **C, E** Colony density was quantified using *ImageJ* software.

Gene expression analysis of 688 colon cancer patient reveals decreased *PTPRO* expression in about 15% of all colon cancers. Down-regulation of *PTPRO* mRNA expression strongly correlates with a poor patient prognosis, highlighting the contribution of PTPRO to colorectal cancer development and progression [14]. To expand this observation, we analyzed PTPRO expression in colon cancer cell lines. qRT-PCR analysis of colon cancer cell lines revealed that the mRNA expression levels of *PTPRO* were dramatically down-regulated in 10 of 14 colon cancer cell lines (Figure 4A). PTPRO protein levels were also reduced in most of the analyzed colon cancer cell lines (Figure 4A), indicating that PTPRO expression is commonly down-regulated in colon cancer cell lines. The observed difference between CRC cell lines and patient data could be due to accumulation of additional alterations during culturing of CRC cell lines [32].

Our results strongly imply PTPRO in negative regulation of EGFR signaling, suggesting that PTPRO expression could affect the sensitivity of colon cancer cells to EGFR inhibitors. Previous reports demonstrated that gefitinib treatment dramatically suppresses EGFR phosphorylation at Y845, whereas gefitinib-resistance is associated with increased SRC-dependent EGFR phosphorylation at Y845 [33–35]. Prior study also proposed that both enhanced SRC activity and EGFR phosphorylation at Y845 could be a potential mechanism of cetuximab resistance in colon cancer [36]. Because we found that PTPRO plays a crucial role in EGFR phosphorylation at Y845, we first examined the effect of PTPRO suppression on survival of LIM1215 cells treated with increasing concentrations of gefitinib. We found that PTPRO depletion (Figure 4B) resulted in resistance to gefitinib treatment in LIM1215 cells (Figure 4C). Strikingly, gefitinib treatment of PTPRO knockdown cells did not affect the levels of SRC phosphorylation at Y416 (Figure 4D). Furthermore, increased activity of SRC in PTPRO-depleted cells led to enhanced phosphorylation levels of both EGFR at Y845 and ERK1/2 even in the presence of gefitinib (Figure 4D), suggesting that the resistance of PTPRO-depleted cells to anti-EGFR treatment could be explained by increased SRC activity and up-regulation of the EGFR/MAPK signaling cascade. To confirm that PTPRO-mediated resistance to EGFR inhibition is SRC-dependent, we treated LIM1215-shGFP and LIM1215-shPTPRO cells with gefitinib alone or in combination with the SRC inhibitor AZD0530. We found that LIM1215 cells expressing either shGFP or LIM1215-shPTPRO demonstrated similar colony formation efficiency when were treated simultaneously by gefitinib and AZD0530, indicating that PTPRO affects the sensitivity of EGFR inhibition by regulating SRC activity (Figure 4E). This result also suggests that tumors with low PTPRO expression may be therapeutically targetable by anti-SRC therapies.

**Figure 5:**
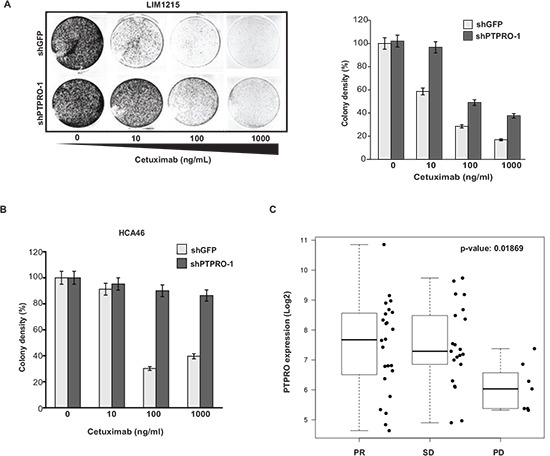
Loss of PTPRO expression leads to resistance to cetuximab **(A)** Colony formation assay of LIM1215 cells expressing shRNAs targeting PTPRO or GFP treated with increasing concentrations of cetuximab. **(B)** Colony formation assay of HCA46 cells expressing shRNAs targeting PTPRO or GFP treated with increasing concentrations of cetuximab. **(C)** The boxplots showing *log2* expression of *PTPRO* gene in the *WT*-*KRAS* population of primary CRC tumors from stage IV patients, according to the best response to cetuximab treatment (PR-partial response, SD - stable disease, PD - progressive disease). **A, B** The results are expressed as mean ± s.e.m. for three independent experiments. **A, B** Colony density was quantified using *ImageJ* software.

Since cetuximab is clinically more relevant to colorectal cancer patients, we also examined whether of PTPRO expression levels affect the response to cetuximab. Cetuximab treatment is currently prescribed only to CRC patients with *WT-KRAS*, therefor*e* we performed the analysis using two WT-KRAS cell lines, LIM1215 and HCA46. In agreement with the results on gefitinib sensitivity, we found that PTPRO depletion in either LIM1215 or HCA46 cells resulted in resistance to cetuximab (Figure 5A, B).

To confirm our *in vitro* results, we next analyzed status of PTPRO expression in CRC and patient response to cetuximab treatment by using a transcriptome-focused approach. We analyzed a cohort of 52 CRC patients with *WT-KRAS* treated by cetuximab in the KULeuven University Hospital [37]. Microarray expression data were normalized using RMA background correction [38] followed by quantile normalization and median polish summarization of probe sets. We applied non-parametric two-sample two-sided Wilcoxon test to compare expression between patients with partial remission (PR) or stable disease (SD) vs progressive disease (PD). Consistently with our *in vitro* data (Figure 5A, B), we observed that CRC patients with progressive disease (PD) have significantly lower PTPRO expression than patients with partial response (PR) and stable disease (SD) (p-value= 0.01869) (Figure 5C). Taken together, our results strongly indicate that PTPRO expression affects the sensitivity of colon cancer cells to EGFR inhibitors. These findings also suggest that PTPRO expression levels may serve as a potential marker to predict response of CRC patients with *WT-KRAS* to EGFR inhibitors.

## DISCUSSION

EGFR activity is determined by the balance of Receptor Tyrosine Kinases (RTKs) and PTP activities. Several PTPs have been recently implicated in tumor suppressors by antagonizing the oncogenic effects of RTK signaling through direct RTK dephosphorylation. A high-throughput loss-of-function screen clearly demonstrated that receptor-type PTPs play a crucial role in negative regulation of major RTKs [39]. The results of this study suggest that PTPRO could affect phosphorylation of EGFR, EphA2, and HER2, whereas two other reports showed that EphA2 and HER2 as direct PTPRO substrates [15, 40].

**Figure 6:**
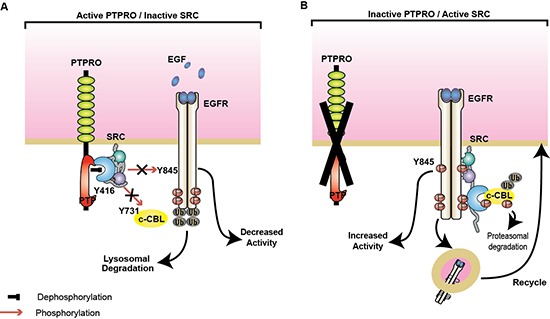
PTPRO negatively regulates SRC/EGFR signaling **(A)** By directly dephosphorylating SRC at Y416, PTPRO inactivates SRC activity that blocks SRC-mediated EGFR phosphorylation at Y845 and c-CBL at Y731. Non-phosphorylated c-CBL triggers EGFR ubiquitination and lysosomal degradation. **(B)** Loss of PTPRO leads to SRC activation and accumulation of EGFR phosphorylated at Y845 and c-CBL at Y731. Phosphorylation of c-CBL promotes its proteasomal degradation and thereby active EGFR is recycling to the plasma membrane.

Our study implicates PTPRO in negative regulation of EGFR signaling through direct inactivation of SRC kinase activity (Figure 6). We observed increased SRC activity in a subset of CRC patients with low PTPRO expression. Importantly, prior studies have reported that elevated SRC activity in CRC is associated with advanced malignancies and metastatic spread [24, 41–44]. SRC contributes to cancer progression by triggering cell proliferation, migration, and invasion. Activated SRC phosphorylates a diverse spectrum of substrates that results in up-regulation of several cancer-associated pathways including EGFR signaling (reviewed in [31, 45]). There is accumulating evidence that the biological synergy between SRC and EGFR promotes colon cancer tumorigenesis [21, 46]. Multiple studies demonstrated that SRC-dependent EGFR phosphorylation at Y845, which is located within the activation loop of EGFR kinase domain, is essential for a full activation of the receptor [18, 20, 21]. EGFR phosphorylation at Y845 has been shown to affect EGFR-dependent growth and migration [47]. Several studies also reported that SRC could also modulate EGFR signaling by phosphorylating and regulating stability of c-CBL, a major EGFR ubiquitin ligase [25]. Here we found that up-regulation of SRC activity by loss of PTPRO dramatically affects activation of EGFR/MAPK pathway, further confirming the contribution of the SRC kinase to colon cancer development and progression.

We also observed that *WT-KRAS* patients with low PTPRO expression are correlated with progressive disease after cetuximab treatment indicating that this group of patients do not benefit from the therapy. Our results revealed that loss of PTPRO promotes resistance to EGFR inhibitors in colon cancer cells by maintaining activated SRC and EGFR/MAPK pathway. Consistently with these observations, it has been reported that EGFR and SRC kinases cooperate in acquired resistance to cetuximab treatment [36, 48]. Increased SRC activity and enhanced EGFR phosphorylation at Y845 has been observed in cetuximab-resistant CRC cells, whereas inhibition of SRC activity sensitizes cells to cetuximab treatment [36]. Furthermore, EGFR phosphorylation at Y845 has been proposed as a diagnostic marker to assess gefitinib sensitivity in colon cancers [49]. Importantly, we found that whereas gefitinib treatment only partially abolished EGFR phosphorylation at Y845, a potent SRC inhibitor AZD0530, completely blocked it. These data suggest that SRC inhibitors might be an alternative therapy for anti-EGFR resistant patients with low PTPRO expression, while several SRC inhibitors are currently being evaluated in multiple clinical trials.

Taken together, our results further highlight the importance of the negative regulation of SRC/EGFR pathway by tyrosine phosphatases. In addition, our results emphasize that a deeper understanding of the regulation of key kinases by phosphatases is crucial for choosing optimal treatment strategy of colon cancer patients.

## MATERIALS AND METHODS

### Cell lines and plasmids

Human colorectal cell lines (HCT15, HCT116, SW480, CACO2, LOVO, LIM1215, DIFI, DKO4, COLO205, HT29, DLD1, HKE3, HCA-46, and HKH2) were obtained from ATCC; HCT15, HCT116, LOVO, COLO205 were cultured in RPMI 1640 medium (Invitrogen); HCA46, SW480, CACO2, LIM1215, DIFI, DKO4, DLD1, HKE3, HKH2, HEK293T were grown in DMEM medium (Invitrogen) and HT29 were grown in McCoy medium (Lonza). All media were supplemented with 10% fetal bovine serum and 1% penicillin/streptomycin. pLKO.1-shPTPRO-1 (TRCN0000002901), pLKO.1-shPTPRO-2 (TRCN0000002903), and pLKO.1-shGFP were purchased from Sigma-Aldrich. Lentiviral infections were carried out as described in TRC protocols (http://www.broadinstitute.org/rnai/public/resources/protocols). Infected cells were selected by treatment with 3 μg/ml puromycin (InvivoGen) for 2 days. Wild type (WT) full length PTPRO expression construct was purchased from ORIGENE. GST-tagged catalytic domain of WT-PTPRO (pGEX-5X-1-WT-PTPRO) plasmid was a kind gift of Dr. Hiroyuki Seimiya, The University of Tokyo. To generate a trapping mutant DA-PTPRO, a point mutation resulting in D1102A mutation, was introduced by using QuickChange Site-Directed mutagenesis kit (Stratagene).

### Immunoblot analysis and immunoprecipitation

Cells were lysed in a lysis buffer (25 mM Tris-HCl Ph7.4, 150 mM NaCl, 1% NP-40, 1 mM EDTA, 5% glycerol) containing protease inhibitor and phosphatase inhibitor cocktails (Roche). Proteins were separated by SDS-PAGE, transferred to PVDF membranes, and immunoblotted. Immunoprecipitation was performed as described elsewhere[16]. Briefly, cells were lysed in lysis buffer (50 mM Tris-HCl pH 7.5, 150 Mm NaCl, 1% NP-40) containing EDTA free protease inhibitor (Roche). Flag-tagged proteins were immunoprecipitated using anti-Flag (M2) agarose (Sigma-Aldrich) and then eluted with 3×Flag peptides according to the manufacture's protocol.

The following antibodies were used: mouse monoclonal anti-Flag (Sigma-Aldrich, M2), anti-GAPDH (Sigma-Aldrich, GAPDH-71.1), anti-vinculin (Sigma-Aldrich, clone hVIN-1), EGFR Antibody FITC (Santa Cruz Biotechnology, 528), 4G10 Platinum Anti-phosphotyrosine (Millipore); rabbit monoclonal anti-EGFR (Cell Signaling, D38B1), anti-p44/42 MAPK (Erk1/2) (Cell Signaling,137F5), anti-phospho-MEK1/2 (S217/221) (Cell Signaling, 41G9), anti-SRC (Cell Signaling, 32G6), anti-phospho-SRC (Y416) (Cell Signaling, D49G4), anti-c-CBL ( Cell Signaling, C49H8); rabbit polyclonal anti-phospho-EGFR (Y845) (Invitrogen), anti-PTPRO (Santa-Cruz Biotechnology, GLEPP1 H-280), anti-phospho-p44/42 MAPK (Erk1/2) (T202/Y204) (Cell Signaling), anti-phospho-c-CBL (Y731) ( Cell Signaling), anti-phospho-SRC (Y527) (Cell Signaling).

Human EGFR Phosphorylation Antibody Array was purchased from RayBiotech Inc. (Norcross, GA). Total cell lysates were incubated with membranes overnight. The detection and relative quantification of proteins were performed according to the manufacturer's protocol.

### Collaborative enzyme enhance reactive ImmunoAssay (CEER)

Cell lysates were prepared according to the manufacturer's protocol and were sent to Prometheus Laboratories Inc. (San Diego, CA) for further analysis.

### *In vitro* substrate trapping assay

Cell pre-treated with 1mM pervanadate were lysed on ice in a lysis buffer (20 mM Tris, pH 7.5, 100 mM NaCl, 1% Triton X-100, 10% glycerol, 5 mM iodoacetic acid, 1 mM orthovanadate and protease inhibitors). 10 mM DTT was then added for 15 min to inactivate iodoacetic acid and orthovanadate. After centrifugation at 14.000g for 15 min, cell lysates were incubated with GST-tagged recombinant PTPRO or GST alone conjugated to GSH sepharose beads overnight at 4°C. The pulled-downed proteins were resolved on SDS-PAGE gel and detected by immunoblotting.

### *In vitro* dephosphorylation assay

The assay was done as described previously [50]. Briefly, equal molar amounts of SRC with either WT-PTPRO or DA-PTPRO were added to the buffer containing 25mM HEPES (pH 7.4), 0.1 mM EDTA, 5 mM DTT. Reaction was carried out under 30°C for 2hrs with gentle agitation. Aliquots were taken out every 15 min and snap-frozen immediately in liquid nitrogen. The proteins were resolved on SDS-PAGE gel and detected by immunoblotting.

### Flow cytometry analysis

Cells were mobilized with enzyme-free cell dissociation buffer (Invitrogen), immunostained with FITC-conjugated anti-EGFR (Santa Cruz, clone 528) or FITC-conjugated isotype mouse IgG2a control (Santa Cruz) antibodies, and analyzed using FACSCanto flow cytometer (Becton Dickinson).

### Colony formation

2×10^4^ cells per well were plated into 6 well plates and allowed to grow for 10 days in the absence and presence of the indicated drugs and then stained with crystal violet. The number of colonies was quantified using ImageJ software.

### Gene expression analysis

Total RNA was isolated using the RNeasy Mini Kit (Qiagen) according the manufacturer's protocol. 1μg of RNA was used for reverse transcription with 220 units of Superscript II Reverse Transcriptase (Invitrogen) that was followed by quantitative RT-PCR using a 7500 Real Time PCR System (Applied Biosystems). PTPRO expression was normalized against 3 housekeeping genes (RPL13A, GAPDH, and UBC) used as reference gene. PTPRO probe (Hs00243097_m1) was purchased from Applied Biosystem. Calibrated normalized relative quantities (CNRQ) were calculated with qBasePlus 1.1 software using target-specific PCR efficiencies calculated from the inter-run calibration standard curves (Biogazelle).

A CRC specific DNA microarray platform (CRC^DSA^) designed to work with formalin-fixed paraffin-embedded (FFPE) tissue was used for expression profiling of 65 FFPE CRC stage IV primary tumor samples from patients treated by cetuximab in the KULeuven University Hospital [37]. 52 WT-KRAS patients were selected for the analysis. Expression data were normalized using RMA background correction [38] followed by quantile normalization and median polish summarization of probesets. Only the most variable probeset was selected as a representative of EntrezGene ID. Non-parametric two-sample two-sided Wilcoxon test was used to compare expression between patients partial remission (PR) or stable disease (SD) vs progressive disease (PD) as best response to cetuximab therapy.

### Statistical analysis

For the experimental data, unpaired student's T test was used. All tests were considered statistically significant for p<0.05.

## SUPPLEMENTARY FIGURE


