# Supplementary material for: The tyrosine phosphatase PTPRO sensitizes colon cancer cells to anti-EGFR therapy through activation of SRC-mediated EGFR signaling
Source: Oncotarget. 2014 Oct 11;5(20):10070–83. doi: 10.18632/oncotarget.2458 (PMC4259406; doi:10.18632/oncotarget.2458)
Supplement: Supplementary file 1 [file oncotarget-05-10070-s001.pdf]

SUPPLEMENTARY FIGURE

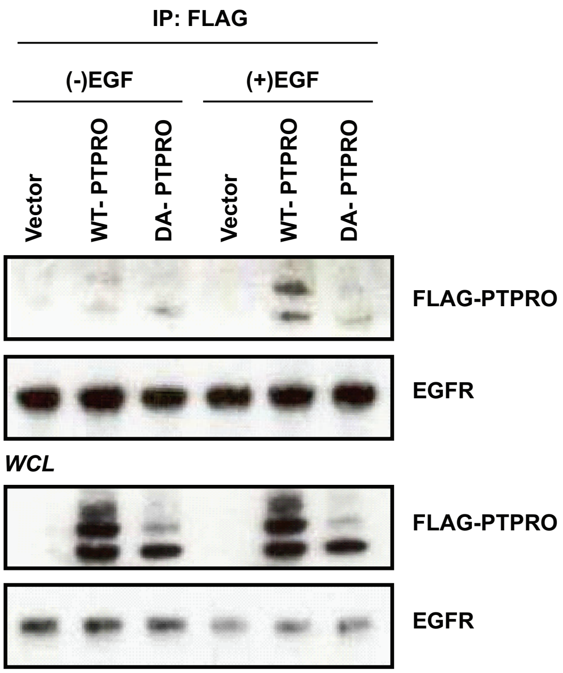

Supplemental Figure S1: PTPRO and EGFR interact in an EGF-dependent manner. An empty vector or Flag-tagged full-length PTPRO (WT and DA) were overexpressed in HEK293T cells. 48 hours after transfection cells were serum starved and then stimulated with EGF (100ng/ml) for 15 minutes. Cell lysates were immunoprecipitated with anti-EGFR antibody and then immunoblotted with antibodies specific to EGFR or Flag.
